# Repeated nitrogen fertilization enhances Scots pine growth and carbon uptake without persistent long-term effects in boreal forests

**DOI:** 10.1093/treephys/tpaf053

**Published:** 2025-05-06

**Authors:** Anni Palvi, Eduardo Martínez-García, Paul Szejner, Katja T Rinne-Garmston, Giles H F Young, Elina Sahlstedt, Raisa Mäkipää, Aleksi Lehtonen

**Affiliations:** Natural Resources Institute Finland (Luke), Latokartanonkaari 9, FI-00790 Helsinki, Finland; Natural Resources Institute Finland (Luke), Latokartanonkaari 9, FI-00790 Helsinki, Finland; Natural Resources Institute Finland (Luke), Latokartanonkaari 9, FI-00790 Helsinki, Finland; Natural Resources Institute Finland (Luke), Latokartanonkaari 9, FI-00790 Helsinki, Finland; Natural Resources Institute Finland (Luke), Latokartanonkaari 9, FI-00790 Helsinki, Finland; Natural Resources Institute Finland (Luke), Latokartanonkaari 9, FI-00790 Helsinki, Finland; Natural Resources Institute Finland (Luke), Latokartanonkaari 9, FI-00790 Helsinki, Finland; Natural Resources Institute Finland (Luke), Latokartanonkaari 9, FI-00790 Helsinki, Finland

**Keywords:** climate change, forest management, forest productivity, intrinsic water-use efficiency, nutrient limitation, wood production

## Abstract

Nitrogen (N) fertilization is known to enhance tree biomass production and carbon (C) assimilation in N-limited boreal forests. Yet, the long-term effects of repeated N applications remain limited. Here, we evaluate the impact of repeated N fertilization at 10-year intervals over six decades on a nutrient-poor Scots pine (*Pinus sylvestris* L.) forest in Central Finland. The analysis encompassed both short-term (single N-addition) and long-term (multi-decadal repeated N-additions) responses of basal area increment (BAI) and carbon isotope composition in tree rings (δ^13^C) from fertilized and control plots from 1960 to 2022. Furthermore, needle mass and chemistry were investigated, and stand-level nitrogen-use efficiency (NUE, amount of stem volume or tree C increased per unit mass of N added) estimated. We found that a single N-addition had a positive short-term effect on the tree ring δ^13^C during the first 2 years after fertilization. This suggests a combined effect on increase in photosynthetic activity and stomatal conductance, likely driven by greater needle mass and higher N content. Basal area increment showed a delayed but rapid increase, attributable to enhanced needle mass from improved photosynthesis, reaching its peak 2 years after fertilization, and then persisting for a period of 4–7 years. However, by the end of each decadal fertilization cycle, BAI and δ^13^C values in N treatment reached those of control, demonstrating no lasting site carry-over effects. The mean decadal NUE for the tree stem volume was 0.16 m^3^ per kg N added, indicating a significant degree of N retention in the forest ecosystem. After six decades, the cumulative impact of repeated fertilization led to a 47% increase in BAI in fertilized plots compared with controls, demonstrating the efficacy of accumulated short-term growth enhancements. Our findings highlight the potential of repeated N fertilization as an effective forest management practice to support the bioeconomy and mitigate climate change in nutrient-poor boreal forests.

## Introduction

The boreal forest region constitutes 27% of the global forested area (Pan et al. 2011), playing a critical role in the global carbon (C) cycle (Bradshaw and Warkentin 2015). However, in many boreal forests, the rate of tree growth is generally constrained primarily by the nitrogen (N) availability (Jarvis and Linder 2000, Binkley and Högberg 2016, Svensson et al. 2023). Nitrogen is a crucial element of amino acids and chlorophyll, making it essential for photosynthesis and growth processes in trees (Millard and Grelet 2010). By adding N, the nutrient availability in the soil increases, enhancing tree photosynthetic activity more efficiently and stimulating biomass production (Brix 1981). It is therefore considered beneficial to fertilize boreal forest stands with N to increase tree biomass production (Mäkipää et al. 1998, Nilsen 2001, Pettersson and Högbom 2004, Bergh et al. 2008, 2014, Jörgensen et al. 2021, Svensson et al. 2023). Since the mid-1900s, N fertilization has been employed commercially in numerous forests of the boreal region through extensive fertilization regimes. Typically, N fertilizers are added to mature forest stands at a standard dose of ~150 kg N ha^−1^, typically one or two times before harvest (Hedwall et al. 2014).

The response of tree growth after single N-addition is related to a range of factors, including site location (Hyvönen et al. 2008), fertility (Saarsalmi et al. 2014), N dose (Hedwall et al. 2014, Kellomäki 2022), tree species and age (Hyvönen et al. 2008), as well as environmental conditions such as growing season temperature and precipitation (Lim et al. 2015). Nonetheless, the effect of N fertilization on tree growth is transient, exhibiting a rapid increase that reaches its maximum in 2–4 years after fertilization (Kellomäki 2022). Subsequently, the effect persists for a period of 6–8 years (Nilsen 2001) before tree growth returns to the levels observed prior to fertilization. This tree growth increase has been linked to short-term modifications in foliage mass and N concentrations (Brix 1981), which can be attributed to alterations in biomass allocation observed in trees after N (Högberg 2007, Poorter et al. 2012). Specifically, the alleviation of N deficiency through fertilization commonly leads to increasing allocation of C to foliage and aboveground woody biomass (Li et al. 2020). This phenomenon can be attributed to an increase in canopy-scale photosynthesis (From et al. 2015, Muhammad et al. 2022), which can be achieved through the increase in foliar N content (Smolander and Oker-Blom 1989), the production of additional leaf area (Cornejo-Oviedo et al. 2017) or a combination of both. However, it should be noted that this positive effect on the canopy-scale photosynthesis may be altered by other limitations induced by foliar deficiencies in other nutrients such as phosphorus and/or lack of light availability due to increased canopy shading (Lim et al. 2015, Tarvainen et al. 2016). Increasing needle mass can also result to dilution effect, where the relative amount of nutrient decreases due to increased leaf area, which can cause nutrient limitations, even though the absolute amount is unaffected (Oren et al. 1988, Tarvainen et al. 2016). The efficacy of N fertilization can also be influenced by water availability, with low precipitation limiting and high precipitation enhancing its effect (Lim et al. 2015).

It has frequently been observed that an extensive fertilization regime in boreal forests results in a relatively minor increase in stem biomass growth, estimated at 10 to 20 m^3^ ha^−1^ with 150 kg N ha^−1^ over a 10-year period, which represents a nitrogen-use efficiency (NUE) of 0.07 to 0.13 m^3^ per one kg of N added (Hedwall et al. 2014). Therefore, the implementation of a long-term multi-decadal repeated N fertilization regime has been suggested to enhance tree growth throughout the entire forest rotation (Kukkola and Saramäki 1983, Bergh et al. 2014, Binkley and Högberg 2016, Jörgensen et al. 2021, Svensson et al. 2023). The application of N fertilization to juvenile trees has been demonstrated to be as beneficial as that to mature trees (Svensson et al. 2023). Furthermore, repeated fertilization can promote modifications to soil processes, potentially leading to residual carry-over effects that may persist over the subsequent forest stand rotations (From et al. 2015). However, repeated fertilization can result in unintended effects, such as alterations to soil microbial community and forest-floor understory diversity (Hedwall et al. 2014), as well as increased risk of N leaching into aquatic ecosystems and ground water resources (Kukkola and Saramäki 1983, Bergh et al. 2008, Lundin and Nilsson 2014, Kellomäki 2022). These concerns have contributed to the limited implementation of this practice in boreal forestry, even though repeated fertilization regimes with small doses have been shown to enhance growth more effectively than a single addition of large amount (Högberg 2007). Consequently, the long-term impact of repeated fertilization regimes on increasing tree biomass production in boreal forests remains uncertain.

The stable C isotopic composition in tree rings (δ^13^C) has been demonstrated to be a useful indicator of N-related changes in physiological processes of trees (Marshall et al. 2022). During CO_2_ diffusion to the chloroplast and subsequent photosynthesis, plants preferentially assimilate the lighter C isotope (^12^C; Farquhar et al. 1982), resulting in δ^13^C variations that reflect environmental conditions and changes in plant physiology (McCarroll and Loader 2004, Brooks and Coulombe 2009, Cornejo-Oviedo et al. 2017). Additionally, the impacts of management practices such as fertilization (Cornejo-Oviedo et al. 2017) and thinning (Lehtonen et al. 2023) have been observed to influence tree level conditions that are reflected in δ^13^C composition in the tree rings. Higher δ^13^C values are typically indicative of increased net photosynthetic rate (*A*_net_), and/or reduced stomatal conductance (*g*_s_) resulting in larger difference between internal and atmospheric CO₂ concentrations, thereby linking δ^13^C closely with environmental variables (Farquhar et al. 1989). For example, higher solar radiation is associated with increased *A*_net_, whereas increases in temperature and water deficit can result in reduced *g*_s_, which represents a protective response aimed at minimizing water loss (Farquhar et al. 1989, Hilasvuori and Berninger 2010, Young et al. 2010, Barisevičiūtė et al. 2017). Consequently, increase in *A*_net_/*g*_s_ translates to higher intrinsic water-use efficiency (iWUE) which relates to the balance between carbon influx per unit change in *g*_s_ (Farquhar et al. 1989, Ehleringer and Cerling 1995, Lambers et al. 2008).

Previous studies have reported an transient increase in δ^13^C after N fertilization (e.g., Betson et al. 2007, Brooks and Mitchell 2011), primarily attributed to greater influence on *A*_net_ (Brix 1981, Jennings et al. 2016, Marshall et al. 2022). Nitrogen fertilization has been observed to increase the N content per unit leaf area, thereby affecting *A*_net_ through enhanced light capture efficiency (Tarvainen et al. 2016) and increased photosynthetic capacity (Smolander and Oker-Blom 1989, Duursma and Marshall 2006, Brooks and Mitchell 2011). While this mechanism can vary between tree species (see, e.g., Munger et al. 2003, Maggard et al. 2016, Zhu et al. 2020), the correlation between *A*_net_ and N content in Scots pine (*Pinus sylvestris* L.) has been found to be significant (Linder and Troeng 1980, Smolander and Oker-Blom 1989, Warren et al. 2003, see also review by Teskey et al. 1994). This relationship suggests that the changes in δ^13^C values observed between N-fertilized and unfertilized trees in Scots pine stands can be primarily attributed to an increase in *A*_net_ due to N-addition (Brooks and Mitchell 2011, Duursma and Marshall 2006) with a proportional change in *g*_s_. Therefore, the increasing leaf N content after N fertilization can be recorded through changes in δ^13^C fixed in the organic matter of these plants.

The available data on δ^13^C in N fertilization experiments in boreal forests remains limited and further evidence is required to elucidate these processes in higher latitudes. This study addresses this knowledge gap by examining the impact of repeated N fertilization at 10-year intervals over six decades on tree growth and C assimilation dynamics (using basal area increment [BAI] and δ^13^C as proxies, respectively) at a N-poor forest stand dominated by Scots pine in Central Finland. Specifically, we explore both the short-term (single N-addition) and long-term (multi-decadal repeated N-additions) responses of BAI and δ^13^C in earlywood (δ^13^C_EW_) and latewood (δ^13^C_LW_) obtained from intra-annual subsections of the tree rings in paired fertilized and control plots over the period from 1960 to 2022. Furthermore, needle mass and chemistry were investigated in both the year and 3 years following the N-addition in 1980 and 1983, respectively. The analysis of successive stand-level measurement data, collected at 10-year intervals from 1980 to 2020, facilitated the estimation of NUE (i.e., the amount of tree C or stem volume increase per unit mass of N added), which was used as a benchmark for the N treatment. The objective of this study was to determine the effects of short- and long-term N-addition on the temporal dynamics of growth and C assimilation at the tree and stand level.

## Materials and methods

### Study site and experimental design

**Figure 1:**
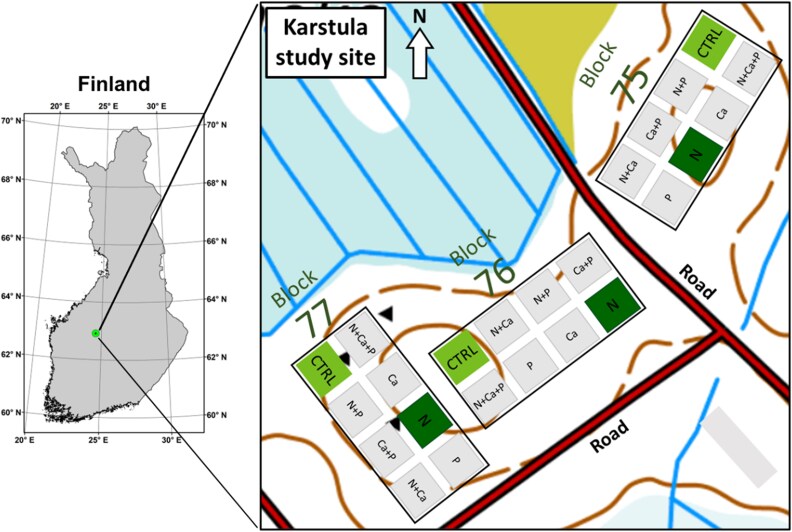
Location and experimental design of the Karstula study site. The spatial distribution of the control (CTRL) and N-fertilized (N) plots within the three study blocks (75, 75 and 77) is shown. Map also shows contour lines, ditches and peatlands. Note that the block and plot sizes are not to scale and for illustrative purposes only.

The study site is a 67-year-old managed Scots pine (*Pinus sylvestris* L.) forest stand located in the municipality of Karstula, Central Finland (62°54′N, 24°34′E; Figure 1). The mean annual air temperature and precipitation sum for the study area over the 62-year period from 1960 to 2022 were 3.3 °C and 646 mm, respectively (FMI 2024). The mean annual photosynthetically active radiation for the 21-year period from 2001 to 2022 was 539 W m^−2^ (NASA 2024). Forest-floor vegetation is mainly composed of dwarf shrubs such as lingonberry (*Vaccinium vitis-idaea* L.), heather (*Calluna vulgaris* L. Hull), crowberry (*Empetrum nigrum* L.), mosses and lichens. Thinning operations took place at the study site in 1969, 1990 and 2014.

A factorial fertilization experiment was established in the study site in 1959 (Saarsalmi et al. 2014). The experimental design was a randomized block design comprising eight plots of 25 m × 25 m size, and with a 5 m buffer zone between plots (Figure 1). Each plot was subjected to a different fertilization treatment, comprising the following: control (without fertilization), N, calcium (Ca), phosphorus (P), N + Ca, N + P, Ca + P and N + Ca + P. This design was subsequently confirmed by three block replicates (75, 76 and 77). The present study was focused on the effects of N fertilization. Therefore, only the control and N treatment plots were used in this study, and the data from the three block replicates were combined.

The granular N fertilizer was distributed evenly by hand to the plots. The first application of N fertilizer was conducted in 1960, which corresponded to 82 kg N ha^−1^, followed by the dose of 120 kg N ha^−1^ in 1970 (Table 1). Subsequent N-additions occurred on 10-year intervals, with a N dose of 180 kg N ha^−1^. The applied N dose was slightly higher than standard dose (150 kg N ha^−1^) due to the low fertility of the site. All study plots were in the same successional stage when N-additions were initiated.

**Table 1:** Types and loads of N fertilizer applied in each decadal addition.

Date	Fertilizer	Amount (kg N ha^−1^)
06/1960	Ammonium sulfate	82
05/1970	Urea	120
06/1980	Ammonium nitrate	180
06/1990	Ammonium nitrate	180
05/2000	Ammonium nitrate	180
05/2010	Ammonium nitrate	180
06/2020	Ammonium nitrate	180

The soil at the study site is a sandy and rocky podzol, characterized by its nutrient-poor and dry conditions. Specifically, different soil properties were evaluated and found to be consistent across the control and N treatment (Table 2), thereby ensuring comparability of the results. The stone content exhibited variability across plots, with values ranging from 34% to 63%. However, the mean stone content was found to be identical (44%) in both control and N treatment.

**Table 2:** Soil characteristics for the organic and mineral layers (0–30 cm depth) of each block and designated treatment plots. The term ‘gravel’ means the mass percentage (%) of the 2–20 mm fraction, while ‘stone content’ refers to the volumetric proportion of stones and boulders of > 20 mm diameter in the 0–30 cm layer.

Block	Plot	Organic layer				Mineral layer			Gravel (%)	Stone content (%)
		Depth (cm)	Carbon %	SOC (t C ha^−1^)	Bulk density (g cm^−3^)	Carbon %	SOC (t C ha^−1^)	Bulk density (g cm^−3^)		
75	Nitrogen	5.2	48.3	24.6	0.11	2.2	23.8	0.89	7.6	37
75	Control	4.1	42.3	16.6	0.11	2.3	23.2	0.82	13.5	36
76	Nitrogen	5.6	48.9	27.1	0.11	3.0	23.8	0.75	11.5	54
76	Control	5.4	49.4	19.7	0.09	2.1	22.9	0.88	13.1	34
77	Nitrogen	5.9	50.1	23.8	0.09	1.2	14.0	1.00	7.9	42
77	Control	4.1	46.6	15.0	0.10	1.6	9.4	0.67	17.5	63

### Tree core sampling and preparation

On 3 November 2022, five healthy dominant trees were randomly selected from the 25 m × 25 m area within both the control and N treatment at each block. One 5 mm diameter increment core was extracted at breast height (1.3 m above the ground) with caliper for each tree for the analysis of tree-ring width and intra-annual δ^13^C. In addition, trees were measured for diameter at breast height (DBH; 1.3 m above ground averaging two perpendicular measurements across the trunk), total tree height (Ht) and tree crown base height (Hcb).

The tree increment cores were air-dried and stored in the laboratory. Subsequently, they were immersed in water for at least 30 min, mounted on wooden supports and planed with a microtome (WSL-Core-Microtome; Swiss Federal Research Institute WSL, Switzerland) until the ring boundaries and wood cells were clearly visible under a binocular microscope.

### Tree-ring width measurements and basal area increment estimation

Each tree core was scanned at a resolution of 800 dpi using a color scanner (Epson perfection v700 Photo). Finally, ring widths were measured with a precision of 0.001 mm using the WinDendro™ software (WinDendro Reg 2022a; Regent Instruments Inc., Quebec, Canada).

The tree rings were cross dated, and their annual dating was corroborated using the dplR package (Bunn 2008). The measurements spanned the period from 1961 to 2022. Tree-ring widths were converted to basal area increments (BAIs; mm^2^ tree^−1^ year^−1^) over the study period.

### Stable carbon isotope composition determination

Tree ring samples were collected from the control and N treatment, spanning the years 1980 to 2022. Individual growth rings from each tree were divided into earlywood (EW) and latewood (LW) using a scalpel under a binocular magnification. Both the EW and LW samples for each year were proportionally pooled from the five trees in each control and N treatment plot. The pooling method maintained consistent mass per subsection of the tree ring when combined, avoiding mass imbalances by larger rings (Belmecheri et al. 2022), resulting an annual composite sample for EW and LW for each plot. This approach allowed us to obtain δ^13^C_EW_ and δ^13^C_LW_ chronologies for both the control and N treatment.

In total, 516 individual tree ring subsections were analyzed (i.e., 43 years [1980 to 2022] × 2 plots [control and N] × 2 tree ring subsections [EW and LW] × 3 blocks [75, 76 and 77]). It is important to note that only 65 of the examined tree rings were too narrow (i.e., width < 0.5 mm), making it difficult to separate the EW and LW components with accuracy. In these cases, the entire ring was analyzed and the same δ^13^C value was applied to both EW and LW components.

After pooling EW and LW components, all the sub-annual samples were extracted to α-cellulose using standard methodologies (Loader et al. 1997, Rinne et al. 2005, Helle et al. 2022). Following the extraction of cellulose, each sample was subjected to homogenization using an ultrasonic probe (Laumer et al. 2009), after which it was freeze-dried. Subsequently, 0.30–0.35 mg of each homogenized sample were weighed into tin capsules and stored in a desiccator cabinet prior to isotope analysis.

The carbon isotope composition of the α-cellulose samples was measured using an elemental analyzer coupled to an isotope ratio mass spectrometer (Europa EA-GSL and 20–22 IRMS, respectively; Sercon Ltd, Crewe, UK) at the Stable Isotope Laboratory of Luke (‘SILL’). The carbon isotope composition (δ^13^C) of earlywood (δ^13^C_EW_) and latewood (δ^13^C_LW_) were determined in parts per mill (‰) relative to the international Vienna Pee Dee Belemnite (V-PDB) standard (Eq. (1)):


(1) \begin{equation*} {\mathrm{\delta}}^{13}\mathrm{C}=\left(\frac{R_{\mathrm{sample}}}{R_{\mathrm{standard}}}-1\right)\times 1000 \end{equation*}

where *R*_sample_ and *R*_standard_ are the ^13^C/^12^C ratios in a sample and standard, respectively. Subsequently, the results were calibrated against IAEA-CH3 (−24.72‰), IAEA-C7 (−32.15‰) and in-house sucrose (−12.22‰) and lactose (−24.66‰) reference materials. Measurement precision was 0.1‰, based on the analysis of multiple measurements (*n* = 14) of a quality control material. Finally, δ^13^C_EW_ and δ^13^C_EW_ values were corrected by the declining δ^13^C levels in atmospheric CO_2_ (McCarroll and Loader 2004).

### Needle dry mass and N concentration

As part of the investigation into the effects of N fertilization on Scots pine, the needle chemistry over two distinct years, 1980 and 1983, were analyzed, corresponding to the year of N fertilization and 3 years later over a single N-addition period. In 1980s, five sample trees were randomly selected from N treatment and control within each of study block. Three south-facing branches, comprising both the current and 1-year shoots, were harvested from the upper third of the crown of each sample tree. Then, shoots with different needle cohorts were separated for each branch and tree in the lab. A composite sample was made for each tree, comprising shoots of the same needle-year cohort. Subsequently, each sample was partitioned into two subsamples. Subsample ‘A’ was composed of three shoots per tree and needle-year cohort and was employed for the determination of the dry needle mass. Subsample ‘B’ was comprised of the remaining shoots and was used for the assessment of N concentration. Ten needles were removed from each subsample ‘A’ and oven-dried at 105 °C for 24 h. Subsequently, needles were weighed, and the weight converted to a 1000-needle basis. Shoots from each subsample ‘B’ were oven-dried at 40 °C for 10 days, after which the needles were removed from the shoots. Subsequently, the needles were grounded using an ultra-centrifugal mill (model ZM-1, Retsch GmbH, Haan, Germany), with a 1-mm mesh screen. The N concentration (%) was then determined spectrophotometrically on the subsamples digested by the Kjeldahl method (Halonen et al. 1983). The dry needle mass and N concentration values of each needle-year cohort were then averaged for each tree.

### Stand measurements and NUE

Successive stand measurements were conducted in 1980, 1990, 2000, 2010 and 2020, during which DBH, Ht and Hcb were recorded for each tree within both the control and N treatment at each block. Estimates were made for both aboveground (i.e., stem, branches and foliage) and belowground (i.e., stump and roots thicker than 1 cm) tree biomasses for each inventory. This was done using Scots pine specific allometric equations as a function of DBH and Ht (Repola 2009). These were then converted to estimate the total tree carbon stock (tree C), assuming that the carbon content of dry biomass was 50%. Furthermore, the above- and belowground tree C stocks removed during the thinning in 2014 were included. Over bark stem wood volume (stem V) estimates were obtained by Scots pine specific taper curve equations defined by Laasasenaho (1982).

To account for the stand-level effect of N-additions, we calculated the NUE for each decade using the tree stand measurements (see Figure S4 available as Supplementary Data at *Tree Physiology* Online). NUE was calculated as the difference in stem V (NUE_stemV_) and tree C (NUE_treeC_) between the control and N treatment, divided by the total amount of N added (see Table 1).

### Statistical analysis

All statistical analyses were performed using R software (version 2023.12.1; R Core Team 2023).

To isolate the effects of the fertilization treatment and account for the effect of tree age and variability in environmental conditions on tree growth and intra-annual δ^13^C values, we reasonably assumed that the trees in all the plots are coetaneous, and the environmental conditions were identical between the N treatment and control plots on each block.

The mean BAI for control and N treatment within each block was calculated from the five sampled trees from each plot. In contrast, only a single pooled sample was available for δ^13^C_EW_ and δ^13^C_LW_ for each plot from each block. Subsequently, the treatment effect was isolated by calculating the residuals of the BAI and δ^13^C values for the period between 1960 and 2022. This was achieved by subtracting the values of the control plot from those of the N treatment of each block. The resulting differences were then averaged to provide a clear measure of the treatment effect while accounting for variability between blocks.

To evaluate the short-term effect of N-addition during a single application, the cumulative BAI (mm^2^ tree^−1^) was calculated for each tree on an annual basis within the 10-year period. The periods were then normalized so that each baseline value was assigned a value of one. Due to missing data for the baseline year in some decades, 28 decades starting in the mid-1960s and two in the mid-1970s were excluded from normalization.

To further evaluate the short-term effect of N-addition, a Superposed Epoch Analysis (SEA, see Chree 1913, Rao et al. 2019) was conducted on the annual BAI treatment effect (period between 1970 and 2022) as well as annual δ^13^C_EW_ and δ^13^C_LW_ treatment effect (period between 1980 and 2022). The SEA enables the comparison of mean lagged values following key events, such as years of N fertilization, thus allowing an assessment of the differences in BAI and δ^13^C values during the 10-year interval of N fertilization in comparison to the expected values in the absence of fertilization.

To test the differences in needle dry mass and N concentration between 1980 and 1983, independent *t*-tests for control and N treatment were conducted. *P*-values were adjusted using the Bonferroni correction method to account for multiple comparisons.

Cumulative BAI was calculated also on an annual basis for each tree during the period between 1960 and 2022 to evaluate the long-term effect of decadal repeated N-addition. The difference in absolute (mm^2^ tree^−1^) and relative (%) cumulative BAI values between the control and N plots at the end of the whole period were estimated.

To evaluate long-term effect of repeated N-additions, decadal means for BAI, δ^13^C_EW_ and δ^13^C_LW_ were calculated. In addition, decadal mean treatment effect was calculated from the annual residuals (N treatment minus control). The analysis focused solely on complete decades, including data from the 1970s to the 2010s for BAI, and from the 1980s to the 2010s for δ^13^C_EW_ and δ^13^C_LW_. Differences between treatments were evaluated using a one-way ANOVA for the data with N treatment and control, and to assess differences across decades, treatment effect was tested with one-way ANOVA. When significant differences were found, Tukey’s HSD was applied as a post-hoc test. Assumptions of normality and homogeneity of variances were checked using the Shapiro–Wilk test.

To test the differences in DBH, total height and height of the living crown, *t*-test was applied, and *P*-values were adjusted with Bonferroni correction method.

The correlation between NUE_stemV_ and NUE_treeC_ was analyzed. To find out the statistically significant differences in both NUE_stemV_ and NUE_treeC_ among the 1980s, 1990s, 2000s and 2010s, one-way analysis of variance (ANOVA) was employed with Tukey’s HSD as a post-hoc test.

## Results

### Temporal variations in BAI and δ^13^C during 1960–2022

**Figure 2:**
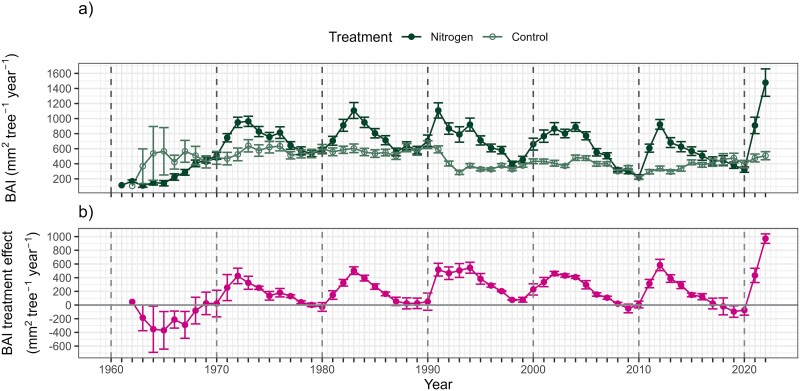
Annual variation in (a) BAI and (b) BAI treatment effect during 1960–2022. Basal area increment treatment effect is calculated as the residuals of control and N treatment for different replicants. The circular symbols indicate annual means of BAI and BAI treatment effect, while whiskers indicate standard errors of the means (BAI: before 1972 *n* = 2–15, 1972 and after *n* = 15; treatment effect: *n* = 3). Dashed vertical lines indicate the years of N fertilization.

The BAI in the N treatment exhibited a consistent increase after each fertilization event, subsequently declining and ultimately reaching control levels before the end of each decade (Figure 2a). In contrast, the BAI in the control plot remained relatively stable throughout the study period, exhibiting minimal fluctuations. The only exception to this decadal pattern was the initial decade of 1960s, when no clear effect of N fertilization was observed in BAI. Consequently, the treatment effect on BAI, calculated as the residuals between N and control, followed a similar pattern that that at the N treatment (Figure 2b). During the 1960s, the BAI treatment effect ranged from −1018 to 252 mm^−2^ tree^−1^ year^−1^, with a mean and standard deviation (SD) of −198 ± 345 mm^−2^ tree^−1^ year^−1^. After this (1970–2022), the BAI treatment effect increased, ranging from −368 to 1059 mm^−2^ tree^−1^ year^−1^, with a mean and SD of 231 ± 235 mm^−2^ tree^−1^ year^−1^.

**Figure 3:**
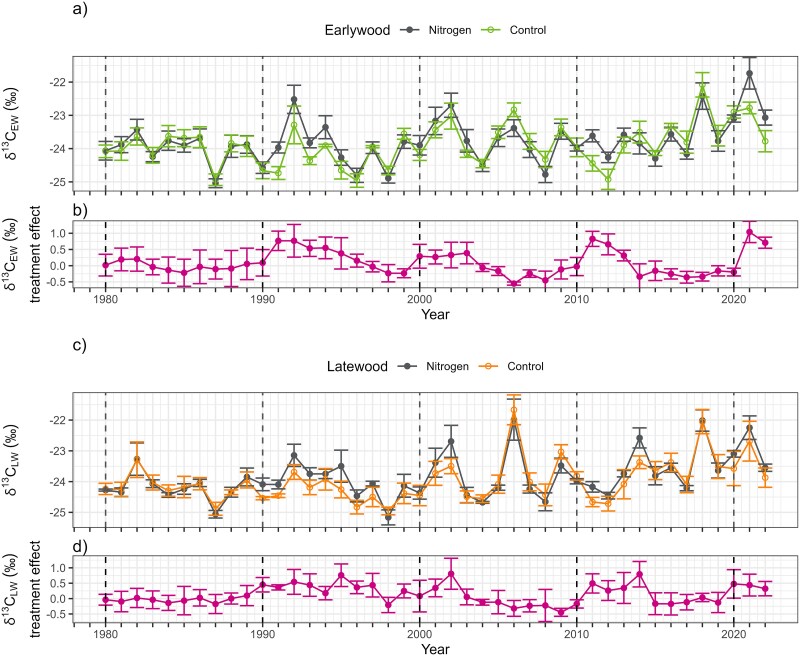
Annual variation in (a) δ^13^C_EW_ and (b) δ^13^C_EW_ treatment effect as well as (c) δ^13^C_LW_ and (d) δ^13^C_LW_ treatment effect during 1980–2022. The treatment effect is calculated from the residuals of control and N treatment for different replicants. The circular symbols indicate annual means of δ^13^C and δ^13^C treatment effect, calculated as the difference between δ^13^C in control and N plots. Whiskers indicate standard errors of the means (*n* = 3). Dashed vertical lines indicate the years of N fertilization.

During 1980–2022, both the N treatment and control showed notable inter-annual variations in δ^13^C_EW_ (Figure 3a) and δ^13^C_LW_ (Figure 3c). These δ^13^C values reflected prevailing environmental conditions, as indicated by their significant correlations with SPEI (δ^13^C_EW_  *r* = −0.365, *P* < 0.001; δ^13^C_LW_  *r* = −0.497, *P* < 0.001; see Figure S1 available as Supplementary Data at *Tree Physiology* Online) and PAR (δ^13^C_EW_  *r* = 0.569, *P* < 0.001; δ^13^C_LW_  *r* = 0.701, *P* < 0.001; see Figure S2 available as Supplementary Data at *Tree Physiology* Online). Although the overall influence of N fertilization on δ^13^C was relatively small in relation to the variability of the δ^13^C records (Figure 3a and c), it was still discernible. Furthermore, the treatment effect (i.e., residuals that isolate fertilization from environmental variation) revealed a clearer pattern over each 10-year fertilization interval. The δ^13^C_EW_ treatment effect ranged from −1.17 to 1.64‰, with a mean and SD of 0.09 ± 0.58‰ (Figure 3b), while the δ^13^C_LW_ treatment effect ranged from 0.95 to 1.64‰, with a mean and SD of 0.13 ± 0.55‰ (Figure 3d). Moreover, in later decades when the trees were older, fertilization appeared to have an increasingly positive effect on δ^13^C.

### Short-term effect of N-addition in BAI, δ^13^C and needle properties

**Figure 4:**
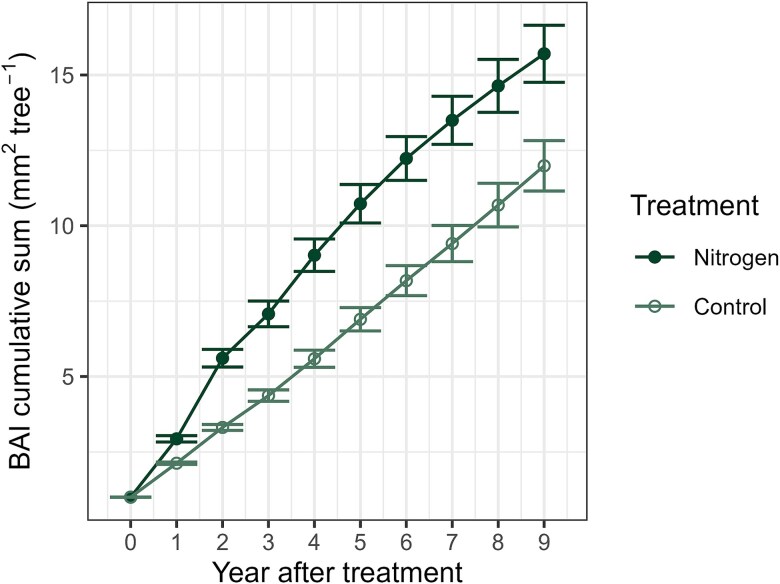
Cumulative BAI sum for the N treatment and control over the years following the fertilization event. The circular symbols indicate the means, while the whiskers represent the standard error of the mean (year 0–2 *n*_Nitrogen_ = 90, *n*_Control_ = 88; year 3–9 *n*_Nitrogen_ = 75, *n*_Control_ = 73). The data for each year and treatment were normalized and averaged over the five studied decades (1970s to 2010s).

The cumulative normalized BAI sum revealed a 7-year sustained increase in growth rates between the N treatment and control (Figure 4). Initially, the growth difference increased rapidly following the fertilization event, with the gap steadily expanding and reaching its maximum at a difference of 4.09 in the seventh year (see Table S1 available as Supplementary Data at *Tree Physiology* Online). Subsequently, the growth differential between the N treatment and control started to decrease, indicating a slowdown in tree growth within the N treatment.

**Figure 5:**
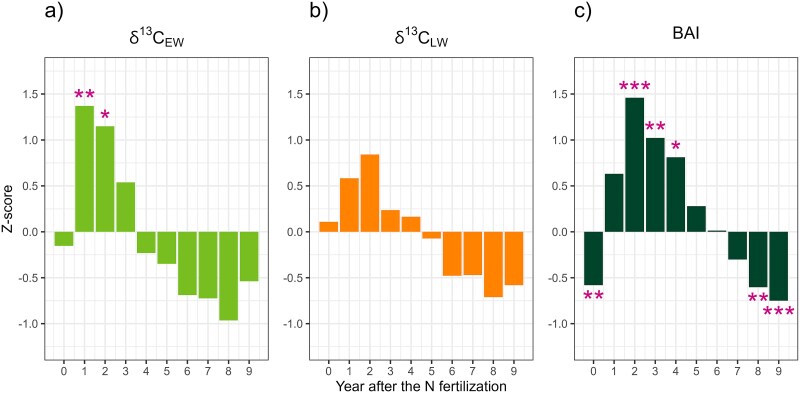
Superposed epoch analysis testing the significance of the short-term effect of N fertilization on (a) δ^13^C_EW_, (b) δ^13^C_LW_ and (c) BAI over the 10-year interval. Positive and negative values indicate values above and below the average of 10-year period. Year 0 in *x*-axis represents the year of N fertilization. For each panel, asterisks represent significant differences between years (^*^P ≤ 0.05, ^**^P ≤ 0.01, ^***^P ≤ 0.001).

The results of the SEA analysis demonstrate an increased δ^13^C_EW_ values in the first (SEA *Z* = 1.37, *P* < 0.001) and second (SEA *Z* = 1.15, *P* = 0.007) years following N fertilization, relative to the mean δ^13^C_EW_ value (Figure 5a). However, by the third year, the increase no longer reached statistical significance (SEA *Z* = 0.53, *P* = 0.081). This finding indicates that the initial enhancement in δ^13^C_EW_ is pronounced but transient, with a rapid increase followed by a gradual decline. In contrast, although the δ^13^C_LW_ values exhibited a similar upward trend during the first 2 years after N fertilization (Figure 5b), none of the differences from the mean were statistically significant. The δ^13^C_LW_ peaked in the second year (SEA *Z* = 0.84, *P* = 0.085), which was followed by a return to the baseline levels by the third year.

Basal area increment values demonstrate a slower yet longer response to N fertilization compared with δ^13^C, showing a noticeable 1-year lag in impact (Figure 5c). In the first year after N treatment, the increase is discernible but compared with the mean BAI value the effect does not reach statistical significance (SEA *Z* = 0.63, *P* = 0.101). Subsequently, a significant yet gradually diminishing effect on BAI is observed in the second (SEA *Z* = 1.46, *P* < 0.001), third (SEA *Z* = 1.02, *P* = 0.004) and fourth (SEA *Z* = 0.81, *P* = 0.021) years following N fertilization. Beyond this period, the BAI values rapidly return below the mean levels observed at the onset of the N fertilization, indicating that the influence of N fertilization on tree growth is also transient. Nevertheless, the effect persisted for a considerably longer period than that observed with δ^13^C_EW_ and δ^13^C_LW_.

**Figure 6:**
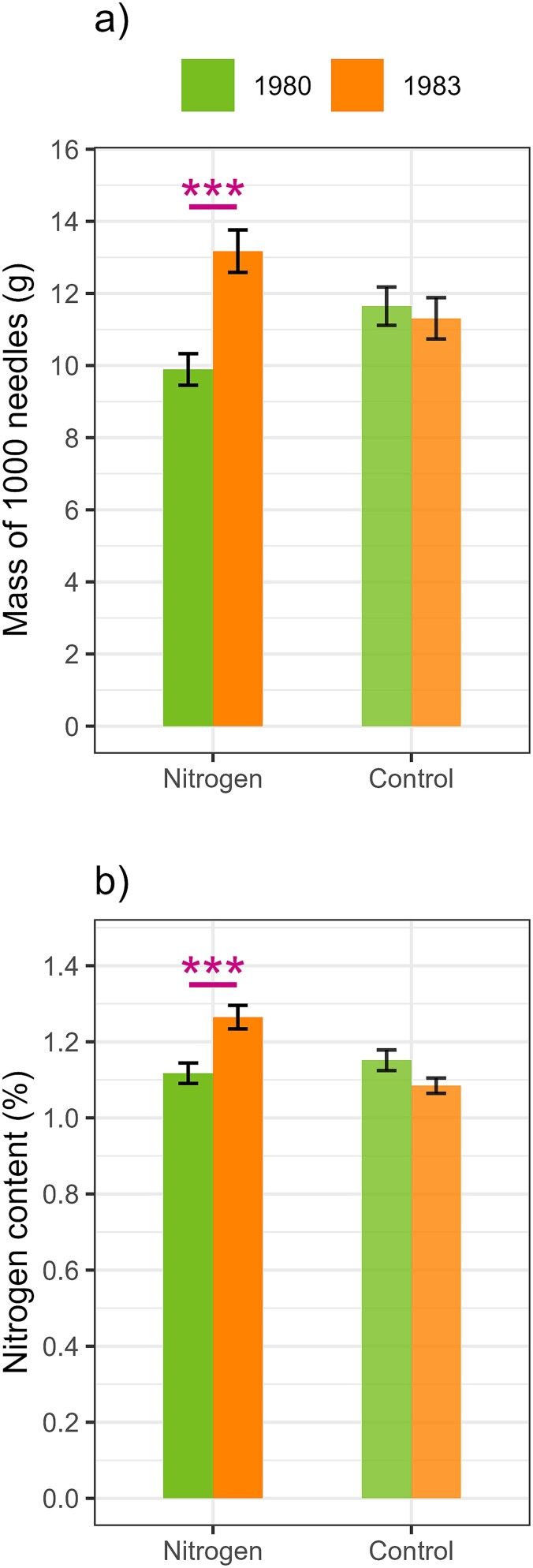
Needle (a) dry mass and (b) nitrogen (N) concentration for the N treatment and control in the fertilization year (1980) and 3 years after fertilization (1983). Needle dry mass is expressed as 1000-needle basis. Whiskers represent the standard error of the mean (*n* = 15). Asterisks represent significant differences (^*^P ≤ 0.05, ^***^P < 0.001) between the years.

The analysis of needle properties revealed variations in both needle mass (Figure 6a) and relative N content (Figure 6b). Specifically, the needle mass exhibited a 52.5% increase in the N treatment from year 1980 to 1983 (*t* = −4.71, *P* < 0.001), while no discernible change was observed in the control (*t* = −0.30, *P* = 0.765). Furthermore, the relative needle N content showed a 17.3% increase in the N treatment from year 1980 to 1983 (*t* = −4.41, *P* < 0.001), while the amount in the control remained stable (*t* = 0.45, *P* = 0.659).

### Long-term effect of repeated N fertilization in BAI, δ^13^C and NUE

**Figure 7:**
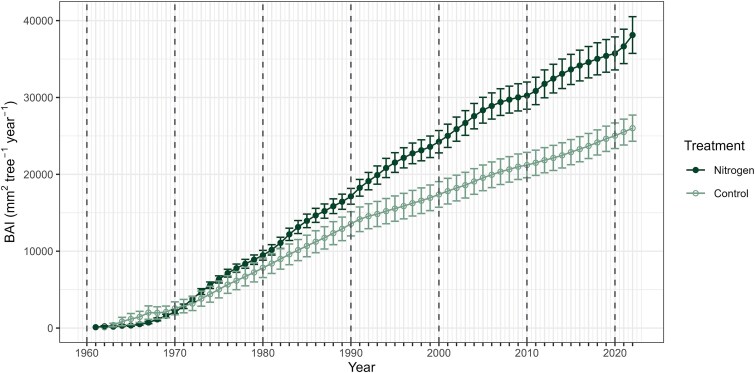
Cumulative BAI sum for the N treatment and control during 1961 to 2022. The circular symbols indicate the means, while the whiskers represent the standard error of the mean (before 1972 *n* = 1–15, after 1972 *n* = 15). Dashed vertical lines indicate the years of N fertilization.

Apart from the initial 1960s decade, the cumulative BAI consistently demonstrated enhanced growth under N treatment in comparison to the control. Following seven fertilization events over six decades, the difference in BAI between the N treatment and control reached its maximum at 12,116 mm^2^ tree^−1^ in 2022, with the N treatment being 46.6% higher than the control (Figure 7). It is important to note that the cored trees at the N treatment not only exhibited larger DBH (*t*_27.2_ = 2.8, *P* = 0.027), but were also taller compared with those at the control (*t*_17.3_ = 4.8, *P* < 0.001; see Figure S3 available as Supplementary Data at *Tree Physiology* Online). Stand measurements from 2020 corroborated this finding (Figure S4c and d available as Supplementary Data at *Tree Physiology* Online).

**Figure 8:**
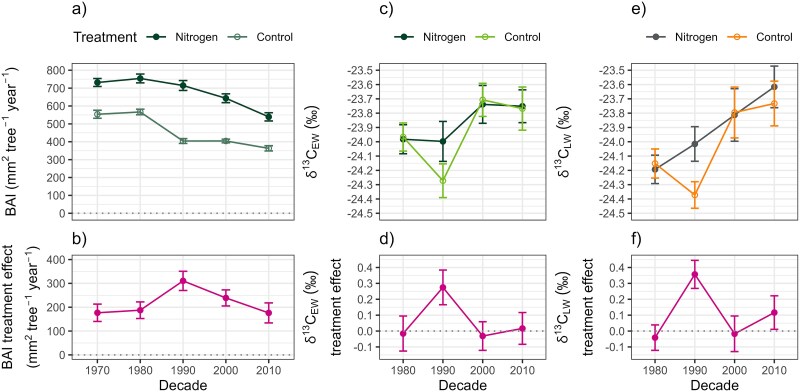
Decadal means for the N treatment and control of (a) BAI and (b) BAI treatment effect, (c) δ^13^C_EW_ and (d) δ^13^C_EW_ treatment effect, as well as (e) δ^13^C_LW_ and (f) δ^13^C_LW_ treatment effect. The treatment effect is calculated from the residuals between control and N treatment in the replicants. The circular symbols indicate the means, while the whiskers represent the standard error of the mean (a: *n* = 148–150, b–f: *n* = 30).

A comparison of the BAI decadal means between the treatments revealed that the N treatment resulted in a significant increase in tree stem growth, with BAI values showing a pronounced response to fertilization compared with the control (Figure 8a; Welch *t*_27.5_ = −5.82, *P* < 0.001). However, decadal BAI appeared to decrease with increasing tree age, although this decline is similar for both N treatment and control. This observation is reflected in the BAI treatment effect, which remained stable at less than 200 mm^2^ tree^−1^ year^−1^, except for the 90s, when the BAI treatment effect peaked due to a more pronounced decrease in the control compared with the N treatment (Figure 8b). Despite the 90s peak, no significant differences in growth patterns were observed between the decades (ANOVA *F*_4,10_ = 1.74, *P* = 0.217), suggesting that repeated N fertilization did not have a long-term effect on tree growth. See Table S2 available as Supplementary Data at Tree Physiology Online for more details.

In contrast to BAI, the δ^13^C decadal means did not exhibit any significant differences between the N treatment and control (Figure 8c: δ^13^C_EW_  *t*_21.9_ = −0.40, *P* = 0.654; Figure 8e: δ^13^C_LW_  *t*_20.2_ = −0.62, *P* = 0.730). While a decline in δ^13^C values in the control during the 1990s was evident in the decadal δ^13^C treatment effect, this variation between decadal means did not reach statistical significance (Figure 8d: δ^13^C_EW_ treatment effect ANOVA *F*_3,0.2_ = 0.28, *P* = 0.837; Figure 8f: δ^13^C_LW_ treatment effect ANOVA *F*_3,0.3_ = 0.54, *P* = 0.665). The lack of statistical significance suggests that repeated N fertilization did not result in a long-term effect on δ^13^C values.

**Figure 9:**
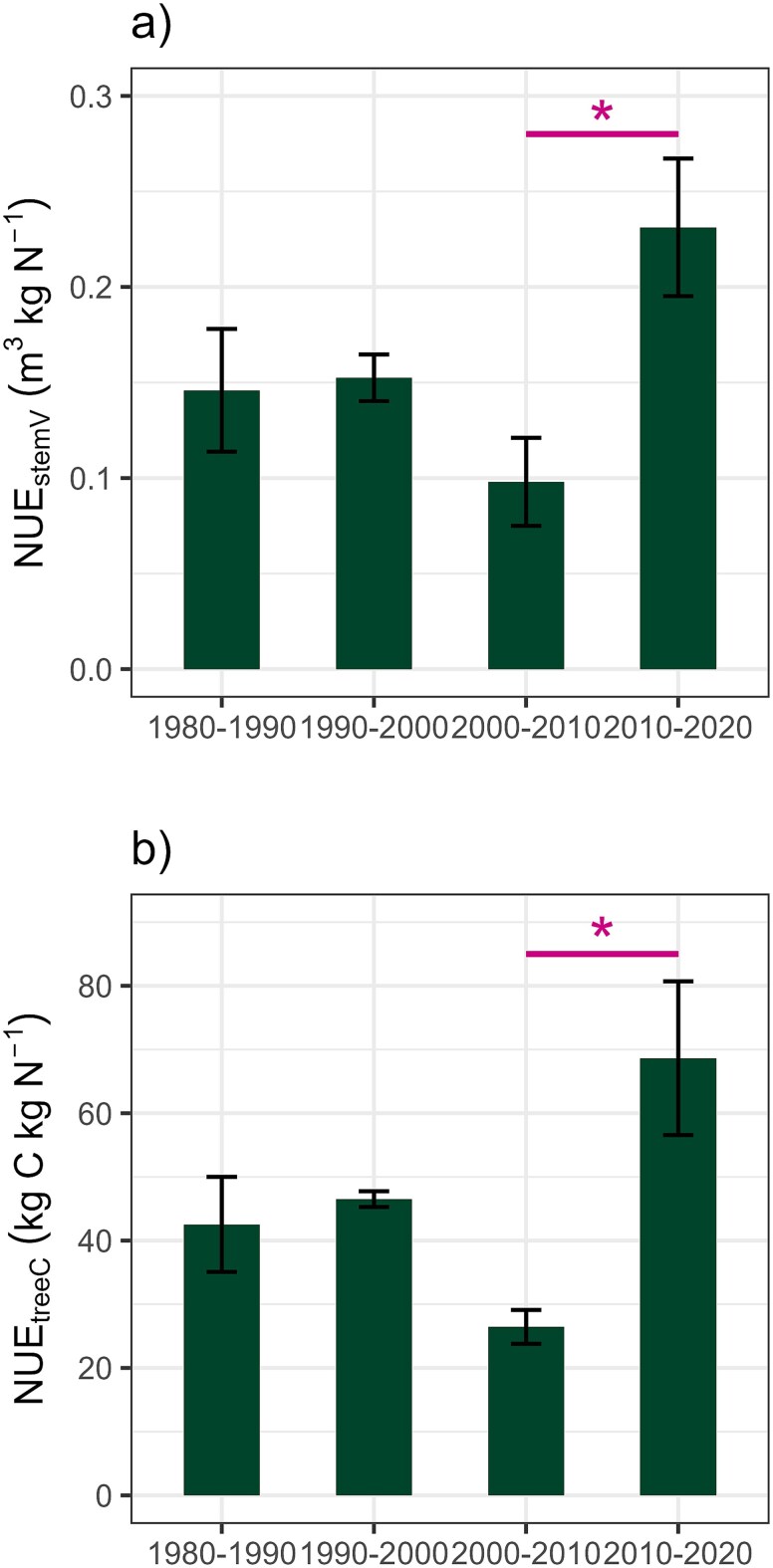
Nitrogen-use efficiency (NUE) showed as the increase in (a) stem wood volume (m^3^) and (b) tree C stock (kg C) per kg of N added for each decade. The removed tree C and biomass in thinning events during 1990 and 2014 has been included in the calculations. The whiskers represent the standard error of the mean (*n* = 3), while asterisks represent significant differences (^*^P < 0.05) between decades.

A strong correlation was found between NUE_stemV_ and NUE_treeC_ (*r* = 0.924, *P* < 0.001, *R*^2^ = 0.854), suggesting that NUE_stemV_ can serve as a good indicator also for the NUE_treeC_. The mean value for NUE_stemV_ for the four decade-period (1980–2020) was 0.16 m^3^ kg N^−1^ (range 0.07–0.30; Figure 9a), while the mean NUE_treeC_ value for the same period was 46.0 kg C kg N^−1^ (range 22.7–91.7; Figure 9b). The NUE differed between the decades (ANOVA: NUE_stemV_  *F*_3,8_ = 4.04, *P* = 0.051; NUE_treeC_  *F*_3,8_ = 5.76, *P* = 0.021), but this disparity was attributable to a great difference between last two decades (Tukey: NUE_stemV_  *diff* = 0.13, *lwr* = 0.01, *upr* = 0.26, *P* = 0.036; NUE_treeC_  *diff* = 42.19, *lwr* = 9.38, *upr* = 74.99, *P* = 0.014). No other significant differences were identified, indicating that repeated N fertilization did not exert a long-term effect on NUE.

## Discussion

### Short-term effects of a single N-addition on tree growth and C assimilation dynamics

Our findings suggest that using a 7-year interval of repeated N fertilization can maximize the tree biomass production (Figure 4). This finding is consistent with previous results in Scots pine stands reporting optimum intervals between 6 and 8 years (Nilsen’s 2001). However, given that the most significant tree growth increase in our study occurred during the years 2–4, shorter N fertilization intervals may be more beneficial in the perspective of tree growth and C accumulation. Supporting our observations, Kukkola and Saramäki (1983) proposed a 4-year fertilization cycle to maximize tree growth and increase profitability. However, considering associated environmental risks with N-addition (Bergh et al. 2008, Pukkala 2017), fertilization amount and interval should be adapted for the site-specific fertility of boreal Scots pine stands.

The analysis of the short-term effects of a single N-addition suggests that the initial responses in δ^13^C_EW_ during the first and second years reflect an increase in iWUE, attributed to the ratio between photosynthetic activity (*A*_net_) and stomatal conductance (*g*_s_). Despite the lack of continuous measurements of soil water availability over the 60-year study period, it can be assumed that both the control and N treatment exhibited equivalent variations in soil moisture values. This assumption was based on the uniformity in soil moisture observed between both treatments in the period 2020–2022 (Figure S5 available as Supplementary Data at *Tree Physiology* Online). Consequently, it can be anticipated that the calculation of δ^13^C_EW_ and δ^13^C_LW_ residuals between control and N fertilization (treatment effect) should remove the environmental effects on *g*_s_. Thus, the observed increase in δ^13^C_EW_ can be largely attributable to the balance of an enhanced *A*_net_, over its physiological relation with *g*_s_, which directly reflects the immediate impact of N fertilization (Jennings et al. 2016). This enhancement is likely attributed to a transient increase in needle N content after N fertilization (Figure 6b), which boosts photosynthetic capacity by increasing the amounts of photosynthetic enzymes and chlorophyll (Linder and Troeng 1980, Smolander and Oker-Blom 1989, Warren et al. 2003, From et al. 2015, Muhammad et al. 2022). This improvement not only enhances the tree’s ability to utilize atmospheric CO_2_, leading to higher δ^13^C values (Duursma and Marshall 2006), but also improves the efficiency of light capture by the needles (Smolander and Oker-Blom 1989). Consequently, the prolonged growth effects observed in BAI might be a consequence of this light capturing efficiency, indicating that the benefits of N fertilization extend well beyond the initial years, enhancing total C assimilation rates.

Another significant factor contributing to tree growth dynamics is the enhanced needle mass after N fertilization (Figure 6a). Basal area increment responses were characterized by an initial growth delay, which was followed by a rapid increase that reached its maximum 2 years after fertilization. This was then followed by a period of 4–7 years where the effect persisted, before tree growth returned to control levels. This tree growth response to N-addition is consistent with findings in Scots pine and Norway spruce in Sweden (Pettersson and Högbom 2004, Kellomäki 2022) and in Douglas-fir in the USA (Brooks and Coulombe 2009). This initial delay in BAI can be attributed to the use of photosynthates for enhanced needle growth during the initial years, which, once established, allows the enhanced C uptake and allocation into the stem wood by the increased leaf area (Cornejo-Oviedo et al. 2017). Although the rate of photosynthesis may decline a few years post-fertilization, the enhanced carbon assimilation via bigger needles can sustain the tree stem growth. However, this enhancement is only temporary, as needles are eventually shed over time. Consequently, in Scots pine the duration of the growth increase by N fertilization correlates with the life cycle of needle biomass (Kellomäki 2022). In this regard, Muukkonen (2005) found that in southern Finland, the average age of Scots pine needles ranges from 2.6 to 3.4 years, extending up to 6 years on fertile sites. In contrast, in Central Finland, where Karstula is located, the average age of the needle cohort is estimated to be 2.5 years. It is also important to note that the increased N availability can further prolong the growing period for evergreen conifers, particularly needles, thereby increasing the *A*_net_ rates (Grossnickle 2000, Iivonen et al. 2001). The gradual decline in BAI over 7 years indicates that although there may be an initial increase in needle mass following N fertilization, this effect is ultimately transient, and once the needles are shed, the total C assimilation rate returns to baseline levels. This hypothesis is further supported by the increased litterfall (Ťupek et al. 2024) and amount of soil organic carbon (Adamczyk et al. 2024) observed in the N treatment at our study site, in addition to the findings from previous long-term fertilization experiments and model simulations (Mäkipää et al. 1998).

In contrast to the pronounced effects on BAI and δ^13^C_EW_, the response of δ^13^C_LW_ values was less marked response and lacked statistical significance. This may be attributable to the higher iWUE in the drier summer months compared with the spring period (Tang et al. 2022). It is known that Scots pine exhibits sensitivity to excessive N fertilization, where water scarcity easily becomes a growth-limiting factor (Högberg et al. 2006, Betson et al. 2007) that can also limit the fertilization effect (Lim et al. 2015). Therefore, this sensitivity to N-addition could be linked to site characteristics. For instance, Cornejo-Oviedo et al. (2017) observed a significant increase in δ^13^C_LW_ during the initial year of N fertilization in moist Douglas-fir stands, which is in contrast to the findings of no significant effect at our dry site in Central Finland. Furthermore, Waszak et al. (2024) found that N fertilization exerts a more pronounced impact on EW than LW in Scots pine at relatively dry sites, where trees frequently exhibit adaptations to maximize water retention, such as reduced stomatal conductance. Consequently, trees in dry sites often show higher iWUE due to stomatal closure, which optimizes carbon gain per unit of water lost (Ehleringer and Cerling 1995, Battipaglia et al. 2013). This functional trait has been shown to conserve water, yet concurrently limit CO_2_ uptake, with the potential to attenuate tree growth rates (Brooks and Coulombe 2009). Conversely, trees in moist locations typically exhibit lower iWUE, as abundant water availability allows them to keep their stomata open, leading to higher rates of transpiration relative to carbon assimilation (Zhou et al. 2013). These physiological responses have the potential to influence not only the overall tree growth rates but also modulate their response to N fertilization. Consequently, it is recommendable to consider site moisture conditions when formulating N fertilization strategies.

### Long-term effects of multi-decadal repeated N-additions on tree growth and C assimilation dynamics

The findings reveal that the repeated N fertilization consistently sustained its benefits across the decades without diminishing in impact, thereby leading to cumulative enhancement in tree growth. Although the tree growth rate gradually decreased over time (Figure 8a), the growth benefit from each N fertilization event was maintained as the trees matured (Figure 8b). This result is consistent with the notion that a greater tree growth response is observed when multiple, low-dose N applications are made in comparison to a single, high-dose application (Högberg et al. 2006, see also Liu et al. 2021). Therefore, it can be assumed that low-dose repeated N fertilization has the potential to maintain tree growth rates throughout the rotation period, while concurrently reducing environmental disadvantages.

We found that following each initial response exhibited by BAI to N-addition, its values invariably returned to the control levels, without any apparent long-term enhancement resulting from a single fertilization event to the subsequent one. This finding underscores the absence of additive benefits from repeated N fertilization across the years. However, it is still noteworthy that although the tree growth values eventually return to control levels, the cumulative impact of repeated N fertilization on tree biomass increase is significant. This highlights that while the specific site conditions may not be improved by N fertilization in the long term, the total tree biomass production over time is significantly enhanced from one fertilization event to another.

Similarly, NUE did not demonstrate any long-term site improvements across the fertilization events. Instead, the findings indicate a relatively elevated NUE (0.16 m^3^ kg N^−1^) in this N-poor Scots pine stand, which contrast with the values observed in extensive fertilization regimes based on N-addition repeated in every 10 years (NUE = 0.07–0.13 m^3^ kg N^−1^; Hedwall et al. 2014) or in N fertilization experiments conducted in juvenile stands in every 1–3 years (NUE = 0.13 m^3^ kg N^−1^; Svensson et al. 2023). This elevated NUE could be indicative of a significant degree of N retention in N-poor sites, so that the negative effects associated with repeated N fertilization (e.g., alterations in soil microbial and forest-floor understory diversity as well as N leaching to watercourses) may be limited. It is also noteworthy that NUE exhibited a remarkable interdecadal variation (see Figure 9), which may be attributed to changes in environmental conditions and water availability, given the substantial influence of precipitation on the effectiveness of N fertilization (Lim et al. 2015). The notable increase in NUE in the 2010s may also be associated with the reduced tree density resulting from thinning conducted in 2014. Moreover, earlier research have shown that more frequent N-additions may lead to reduced variability in NUE (Svensson et al. 2023), which could explain the substantial NUE fluctuation observed in the present study, which was based on N-additions repeated at 10-year intervals.

No signs of N saturation were observed at this nutrient-poor site, suggesting that the lack of response in 1960s may be due to physiological or developmental thresholds of the seedlings, rather than chemical oversaturation of the site. Despite the apparent benefits of fertilizing early in the rotation, no positive response was observed with the initial, relatively low N application (82 kg N ha^−1^). Conversely, the N-fertilized seedlings showed a reduced growth rate compared with the control, suggesting that this early N-addition may not only be unnecessary but potentially detrimental. Alternatively, trees in the N treatment were not only thicker in DBH but also taller, indicating that younger trees might utilize additional N to increase in height rather than in trunk diameter. Furthermore, we found no significant growth differences between trees receiving 120 kg N ha^−1^ in the 1970s and 180 kg N ha^−1^ in the 1980s, suggesting that younger trees may not efficiently utilize higher doses of N. Bergh et al. (2008) reported increased nutrient leaching during the initial fertilization of younger trees compared with more mature trees, supporting the recommendation to delay the start of the fertilization cycle by one to two decades post-planting to allow for natural growth and establishment. This highlights the importance of tailoring fertilization strategies to stand age, species-specific responses and site conditions, as differences in soil fertility and climatic factors significantly influence the effectiveness of N applications. This has also been demonstrated by Boeraeve et al. (2025), who found highly context-dependent fertilization effects in boreal forests.

## Conclusions

This paper emphasizes the important role of repeated N fertilization in the context of climate-smart forestry. While we found no long-lasting carry-over effects between decadal fertilization intervals due to repeated N fertilization, the site continues to respond to additional N inputs without signs of N saturation. After six decades, the BAI was on average 47% higher in the N treatment compared with the control, demonstrating the potential of repeated N-additions to enhance tree C sequestration. Consequently, the relatively high NUE (0.16 m^3^ kg N^−1^) at the study site indicates a significant degree of N retention within the forest ecosystem. In conclusion, our findings highlight the potential of multi-decadal repeated N fertilization as an efficient way to enhance C assimilation in nutrient-poor boreal Scots pine forests.

## Supplementary Material

Supplementary_material_tpaf053

## Data Availability

Tree-ring width and δ^13^C data analyzed in the Stable Isotope Laboratory (SILL) of the Natural Resources Institute Finland (Luke), needle chemistry and forest inventory data and the R code generated in this study are openly available in the Zenodo digital repository at https://doi.org/10.5281/zenodo.15228296.
