# Supplementary material for: Repeated nitrogen fertilization enhances Scots pine growth and carbon uptake without persistent long-term effects in boreal forests
Source: Tree Physiol. 2025 May 6;45(6):tpaf053. doi: 10.1093/treephys/tpaf053 (PMC12166546; doi:10.1093/treephys/tpaf053)
Supplement: Supplementary_material_tpaf053 [file supplementary_material_tpaf053.docx]

Repeated nitrogen fertilization enhances Scots pine growth and carbon uptake without persistent long‑term effects in boreal forests

By Anni Palvi et al.

Correspondence to:

Anni Palvi ([anni.palvi@luke.fi](mailto:anni.palvi@luke.fi))

Contents:

Supplementary Methods

Supplementary Figures S1–S5

Supplementary Tables S1–S2

Supplementary References

# Supplementary Methods

#### 1. Correlations between δ^13^C and environmental conditions

The correlations between the carbon isotope composition (δ^13^C) values and different environmental conditions were analyzed separately for earlywood (δ^13^C_EW_) and latewood (δ^13^C_LW_). For each year, δ^13^C values were compared with corresponding Standardized Precipitation-Evapotranspiration Index (SPEI; see Supplementary Fig. S1) and photosynthetically active radiation (PAR, see Supplementary Fig. S2) data at the Karstula study site. Specifically, SPEI data was sourced from the Global SPEI database by Climatic Research Unit of the University of East Anglia (Climatic Research Unit, University of East Anglia 2024), while PAR data was obtained from Moderate Resolution Imaging Spectroradiometer (MODIS) website managed by the National Aeronautics and Space Administration (National Aeronautics and Space Administration 2023). It is important to note that the analysis for δ^13^C_EW_ utilized mean SPEI and PAR values from April to June, whereas δ^13^C_LW_ deployed mean SPEI and PAR values from June to August.

#### 2. Statistical analysis

Welch's paired t-test adjusted with Bonferroni method was applied to test the differences between control and N treatment for Figures S3, S4, and S5.

# Supplementary Figures


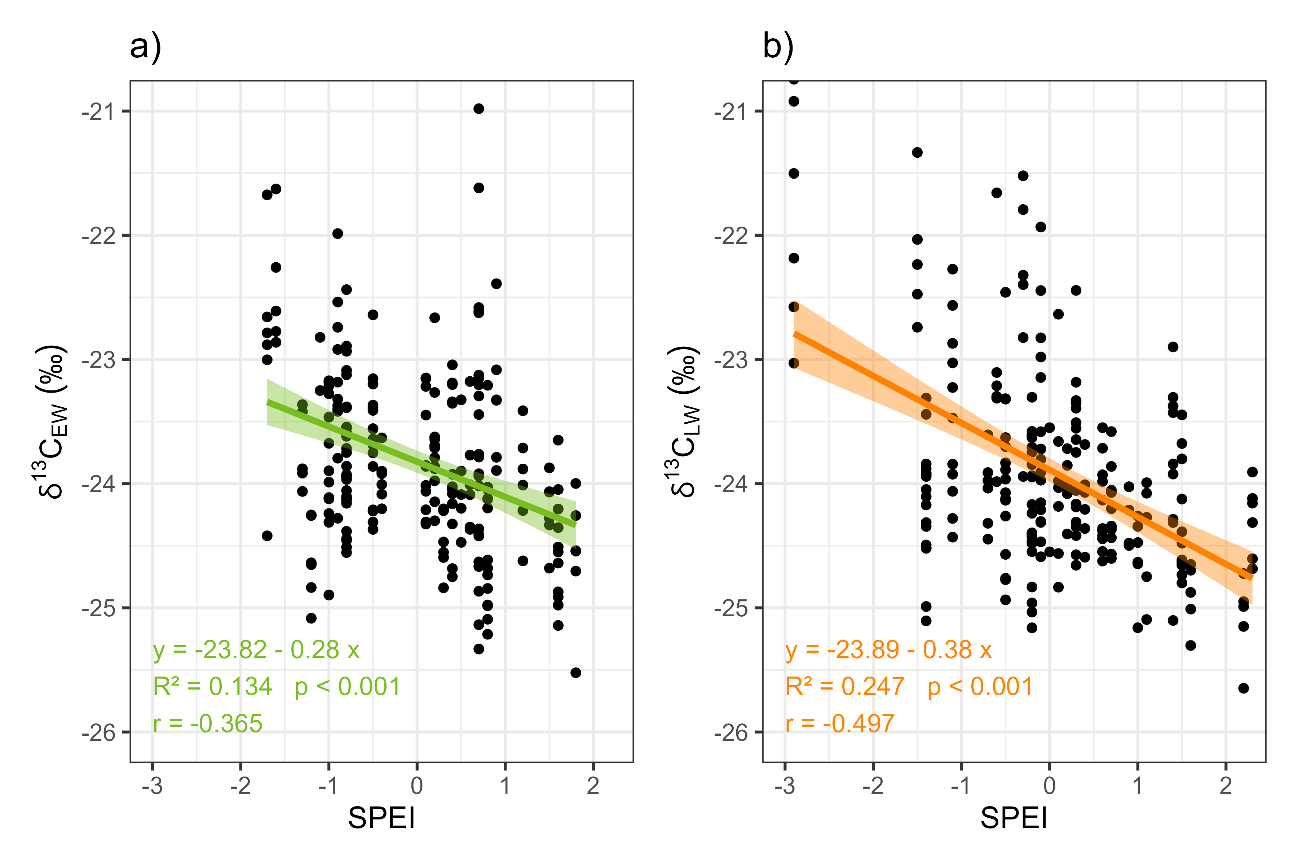


**Figure S1. Correlation between Standardized Precipitation-Evapotranspiration Index (SPEI) and carbon isotope composition of earlywood (δ^13^C_EW_, a) and latewood (δ^13^C_LW_, b).** This figure displays annual data points for each replicant and treatment, aligning SPEI averages from April-May-June for EW and June-July-August for LW.


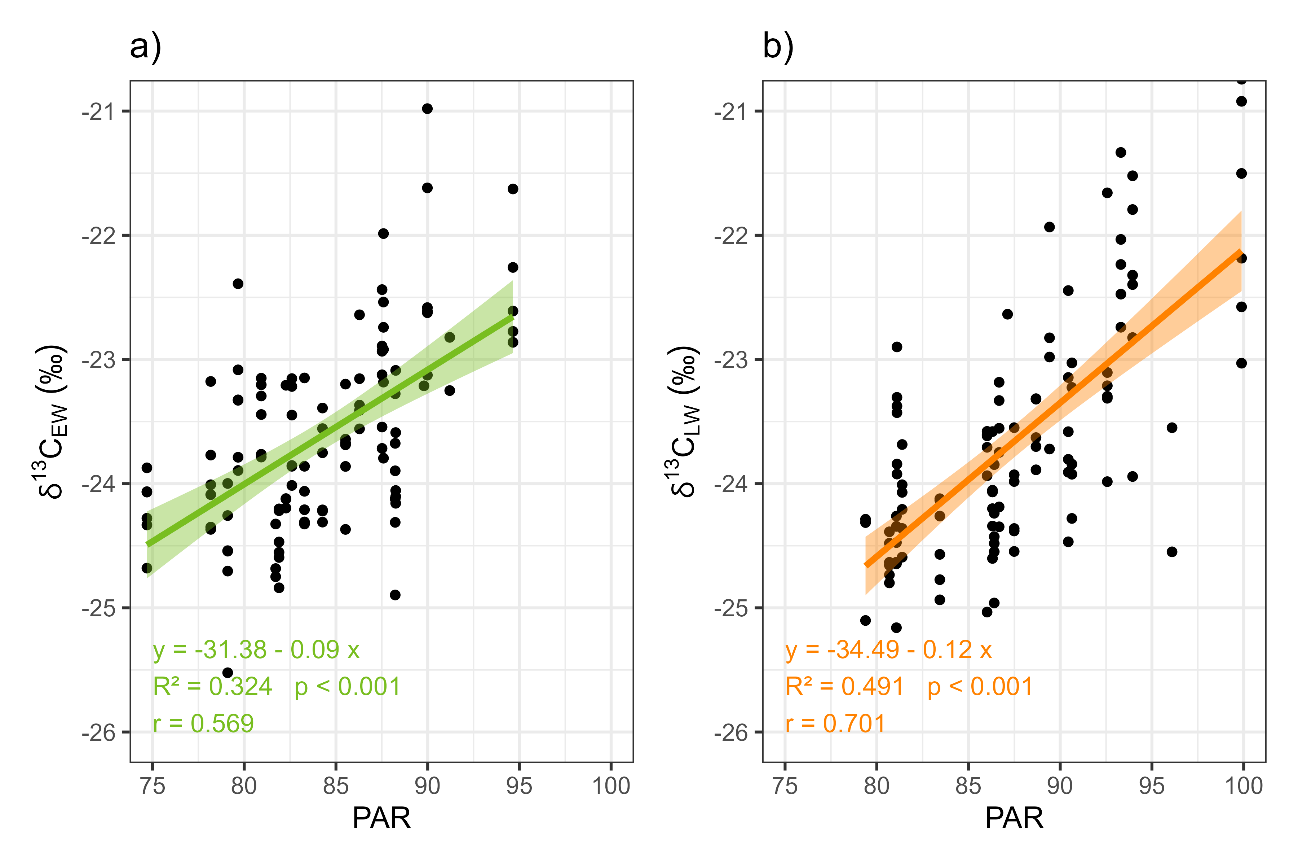


**Figure S2. Correlation between photosynthetically active radiation (PAR) and carbon isotope composition of (a) earlywood (δ^13^C_EW_) and (b) latewood (δ^13^C_LW_).** Each data point represents an annual measurement for each replicant and treatment, using PAR averages from April-May-June for EW and June-July-August for LW.


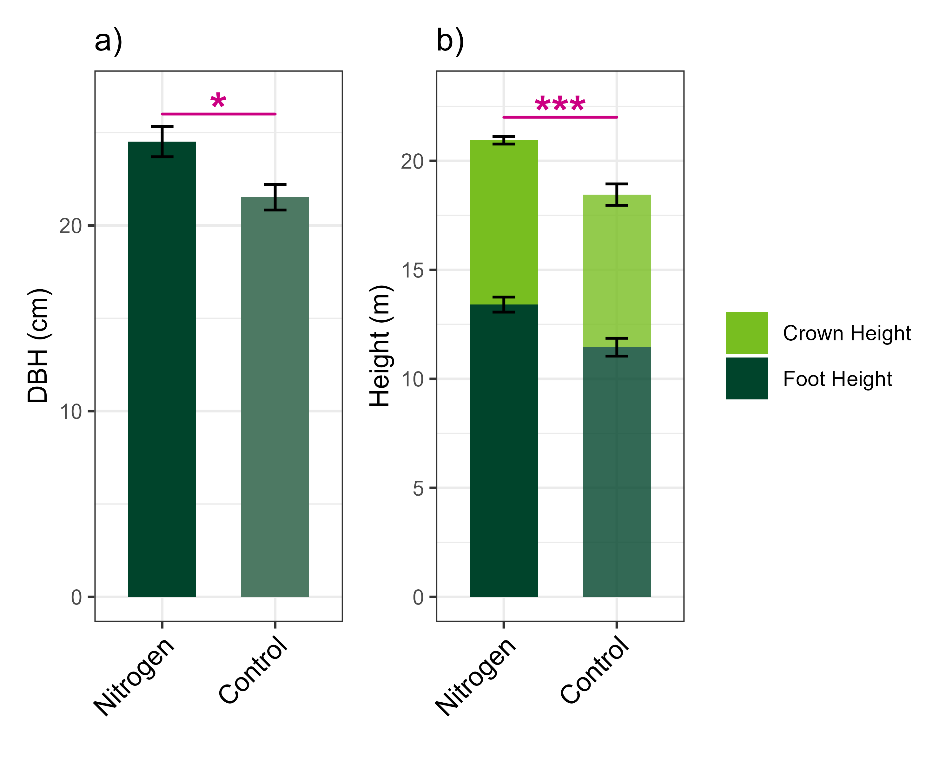


**Figure S3. Statistics of the cored trees at the N treatment and control: (a) Diameter at breast height (DBH; measured 1.3 m above the ground, averaging two perpendicular measurements across the trunk), and (b) total and crown tree heights.** The whiskers represent the standard error of the mean (*n*= 15 trees per treatment), while asterisks indicate significant differences between the treatments (*p ≤ 0.05, ***p ≤ 0.001).


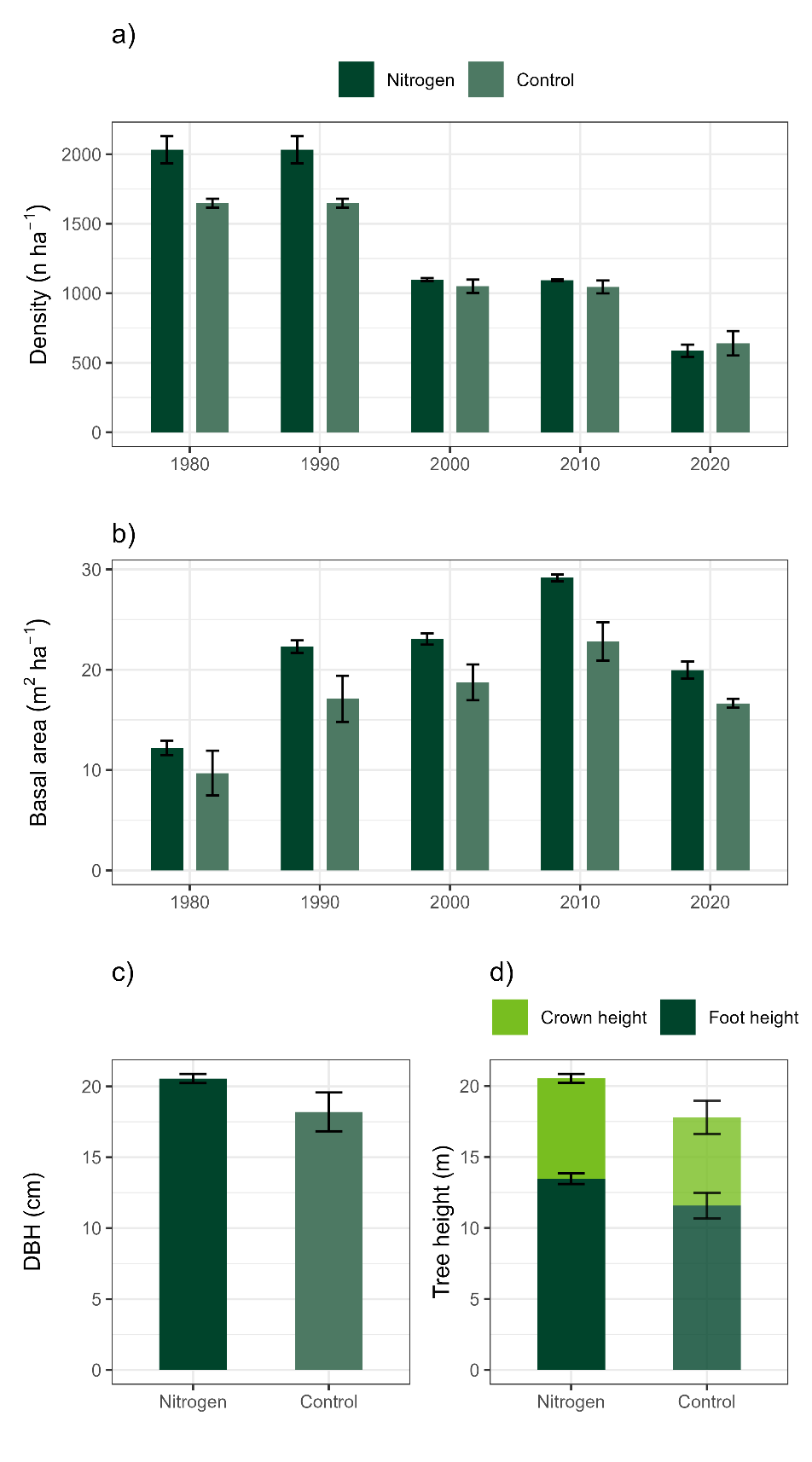


**Figure S4. Statistics of the successive decadal stand measurements conducted in each plot of N treatment and control: (a) Tree density, (b) basal area, (c) diameter at breast height (DBH), and (d) tree height divided in foot and crown.** Tree density and basal area are reported at the beginning of each decade, while DBH and tree heights are reported only for the year 2020. The whiskers represent the standard error of the mean (*n* = 3 plots per treatment). Note that the lack of the statistically significant differences between the N treatment and control is likely attributable to the small sample size.

**
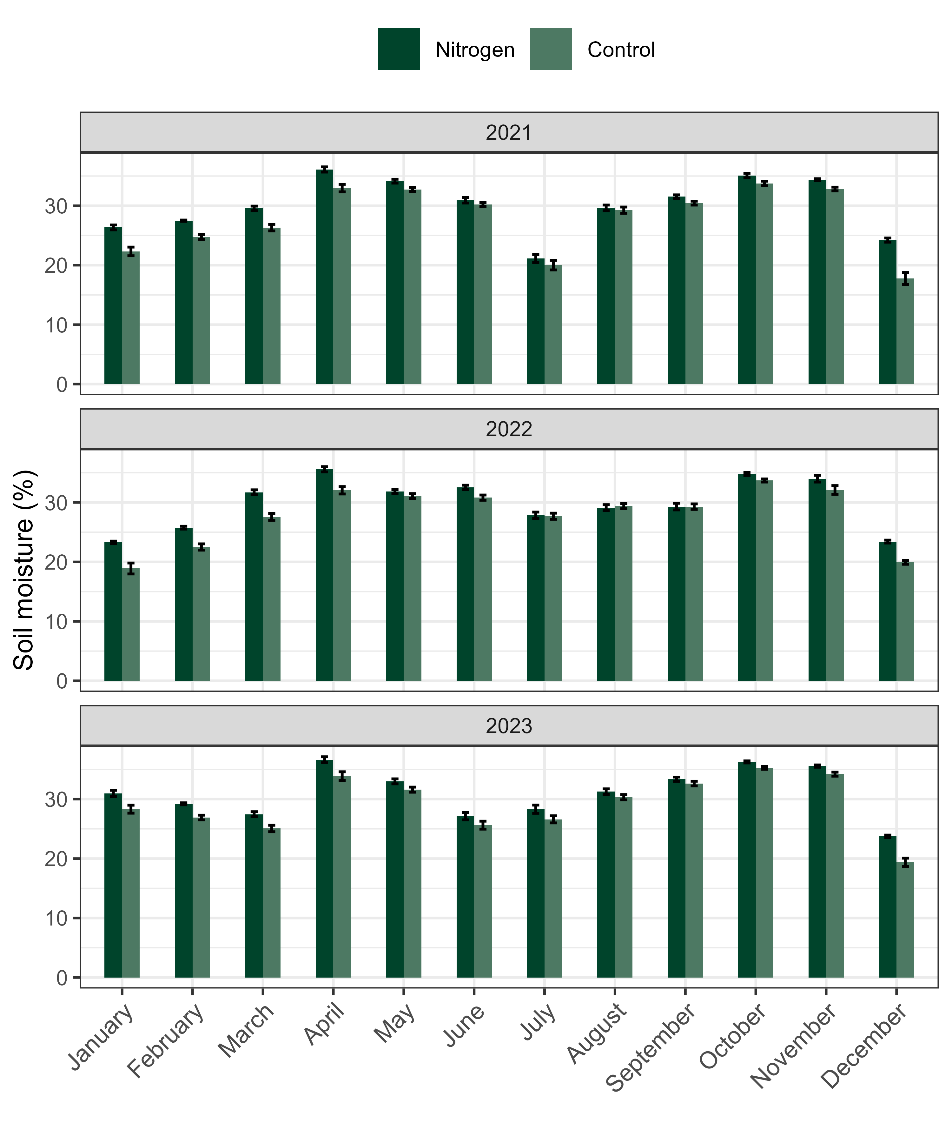
**

**Figure S5. Mean monthly soil moisture at the N treatment and control in years 2021, 2022 and 2023.** The whiskers represent the standard error of the mean (*n* = 72‑124). There were no statistically significant differences found during the growing season months (May to September; *p* = 0.055 – 1, except June 2022 *p* = 0.046). Outside that period (January to April and October to December) the N treatment showed higher soil moisture than control (*p* < 0.049, except October 2021 *p* = 0.296 & 2022 *p* = 0.552 and November 2022 *p* = 0.888). Data were acquired from the Zenodo database (Ťupek et al., 2024).

# Supplementary Tables

**Table S1. Cumulative BAI sum (mm^2^ tree^-1^) for the N treatment and control for each year after fertilization.** Absolute difference in BAI (∆BAI) is also shown.

| **Year after fertilization** | **Control** | **Nitrogen** | **ΔBAI** |
| --- | --- | --- | --- |
| 0 | 1.00 | 1.00 | 0.00 |
| 1 | 2.13 | 2.93 | 0.81 |
| 2 | 3.31 | 5.61 | 2.30 |
| 3 | 4.37 | 7.08 | 2.71 |
| 4 | 5.59 | 9.02 | 3.43 |
| 5 | 6.90 | 10.70 | 3.83 |
| 6 | 8.18 | 12.20 | 4.05 |
| 7 | 9.41 | 13.50 | 4.09 |
| 8 | 10.70 | 14.60 | 3.95 |
| 9 | 12.00 | 15.70 | 3.72 |

**Table S2. Cumulative BAI sum for the N treatment and control for each decade.** The mean cumulative BAI was calculated by averaging the cumulative sum of BAI at the end of each decade (1969, 1979, 1989, 1999, 2009, 2019) for the treatments (N treatment and control). The absolute difference between the N treatment and control was calculated by subtracting the value of cumulative BAI control from that of cumulative BAI N treatment. This difference was further expressed as a relative percentage difference, calculated subtracting cumulative BAI control from cumulative BAI N treatment, dividing the result with cumulative BAI control, and finally multiplying by 100. Absolute increase was calculated by subtracting the absolute difference of the previous decade from the current decade, while relative increase was calculated by subtracting the absolute difference of the previous decade from the current decade, then dividing the result by the absolute difference of the previous decade, and finally multiplying by 100.

| **Decade** | **Cum BAI  N treatment (mm^2^ tree^-1^ year^-1^)** | **Cum BAI control (mm^2^ tree^‑1^ year^‑1^)** | **Absolute difference (mm^2^ tree^-1^ year^-1^)** | **Relative difference (%)** | **Absolute increase (mm^2^ tree^-1^ year^-1^)** | **Relative increase (%)** |
| --- | --- | --- | --- | --- | --- | --- |
| 1960 | 1583 | 2095 | -512 | -24.4 | – | – |
| 1970 | 8898 | 7242 | 1656 | 22.9 | 2168 | 131 |
| 1980 | 16439 | 12908 | 3531 | 27.4 | 1875 | 53 |
| 1990 | 23585 | 16949 | 6636 | 39.2 | 3105 | 47 |
| 2000 | 30018 | 20993 | 9025 | 43.0 | 2389 | 27 |
| 2010 | 35412 | 24625 | 10786 | 43.8 | 1761 | 16 |

# Supplementary References

Tupek, B., Lehtonen, A., Mäkipää, R., & Salovaara, P. (2024). Soil and understory CO2 respiration, CH4, and N2O fluxes, tree biomass and litter, and soil carbon stock after a long-term N fertilization of a Scots pine forest in Finland [Data set md5:a91fb400f7892fb7a90551848f0d646b, accessed 12 December 2024]. Zenodo. <https://doi.org/10.5281/zenodo.13952779>
